# Chitin nanocrystal-assisted 3D bioprinting of gelatin methacrylate scaffolds

**DOI:** 10.1093/rb/rbad058

**Published:** 2023-06-07

**Authors:** Zhengyun Ling, Jian Zhao, Shiyu Song, Shuwei Xiao, Pengchao Wang, Ziyan An, Zhouyang Fu, Jinpeng Shao, Zhuang Zhang, Weijun Fu, Shenghan Song

**Affiliations:** School of Medicine, Nankai University, Tianjin 300071, China; Department of Urology, The Third Medical Center, PLA General Hospital, Beijing 100039, China; Medical School of PLA, Beijing 100853, China; Department of Urology, 960th Hospital of PLA, Jinan 250031, China; Undergraduate Student Majoring in Clinical Pharmacy, Chongqing Medical University, Chongqing 400016, China; Department of Urology, Air Force Medical Center, Beijing 100142, China; Department of Urology, The Third Medical Center, PLA General Hospital, Beijing 100039, China; Medical School of PLA, Beijing 100853, China; Department of Urology, The Third Medical Center, PLA General Hospital, Beijing 100039, China; Medical School of PLA, Beijing 100853, China; Department of Urology, The Third Medical Center, PLA General Hospital, Beijing 100039, China; Medical School of PLA, Beijing 100853, China; Department of Urology, The Third Medical Center, PLA General Hospital, Beijing 100039, China; Medical School of PLA, Beijing 100853, China; Beijing Institute of Basic Medical Sciences, Beijing 100850, China; School of Medicine, Nankai University, Tianjin 300071, China; Department of Urology, The Third Medical Center, PLA General Hospital, Beijing 100039, China; Department of Vascular Surgery, Beijing Chaoyang Hospital, Capital Medical University, Beijing 100020, China

**Keywords:** 3D bioprinting, gelatin methacrylate, chitin nanocrystal, hydrogels, scaffolds

## Abstract

In recent years, there has been an increasing focus on the application of hydrogels in tissue engineering. The integration of 3D bioprinting technology has expanded the potential applications of hydrogels. However, few commercially available hydrogels used for 3D biological printing exhibit both excellent biocompatibility and mechanical properties. Gelatin methacrylate (GelMA) has good biocompatibility and is widely used in 3D bioprinting. However, its low mechanical properties limit its use as a standalone bioink for 3D bioprinting. In this work, we designed a biomaterial ink composed of GelMA and chitin nanocrystal (ChiNC). We explored fundamental printing properties of composite bioinks, including rheological properties, porosity, equilibrium swelling rate, mechanical properties, biocompatibility, effects on the secretion of angiogenic factors and fidelity of 3D bioprinting. The results showed that adding 1% (w/v) ChiNC to 10% (w/v) GelMA improved the mechanical properties and printability of the GelMA hydrogels, promoted cell adhesion, proliferation and vascularization and enabled the printing of complex 3D scaffolds. This strategy of incorporating ChiNC to enhance the performance of GelMA biomaterials could potentially be applied to other biomaterials, thereby expanding the range of materials available for use. Furthermore, in combination with 3D bioprinting technology, this approach could be leveraged to bioprint scaffolds with complex structures, further broadening the potential applications in tissue engineering.

## Graphical abstract

**Figure:**
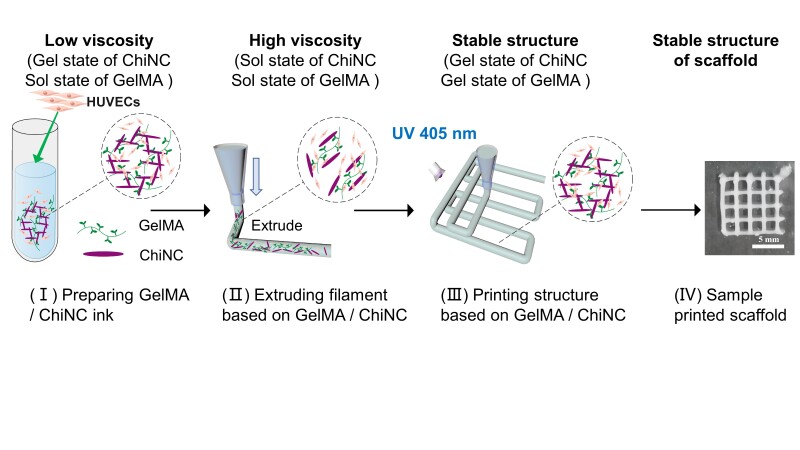

## Introduction

In recent years, there has been a burgeoning interest in hydrogel-based research, with its primary applications encompassing tissue engineering [1–5], cancer models [6–10], drug-controlled release [11–13] and biosensors [14–17]. Among these research areas of study, tissue engineering has emerged as the most prominent direction [18]. Traditional tissue engineering has predominantly utilized medical metals, thermoplastics, bio-ceramics, hydrogels and other materials. Tissue engineering is an emerging discipline that combines cell biology and material science to construct tissues or organs *in vitro* or *in vivo*. Hydrogels, which can mimic the extracellular matrix microenvironment, have demonstrated benefits in promoting nutrient penetration and facilitating cellular communication, migration and growth [19]. These advantages have established hydrogels as a research focal point in the tissue engineering domain in recent years. Despite the numerous benefits of hydrogels, challenges persist in accomplishing complex tissue and organ repair and reconstruction, as traditional methods struggle to accurately repair and reconstruct intricate tissues and organs. Fortunately, the advent of 3D bioprinting technology presents new possibilities for tissue and organ repair and reconstruction [20–22]. The integration of hydrogels with 3D bioprinting technology in tissue engineering offers several advantages over conventional methods. 3D bioprinters can generate micron-level structures with high precision and facilitate personalized customization. Moreover, 3D bioprinting technology can create complex structural models that are challenging to accomplish using traditional techniques. This technological advancement may introduce novel approaches and hold significant clinical practical value for the repair and reconstruction of tissues and organs.

Numerous studies have been conducted on hydrogels for 3D bioprinting [23–26]. In our study, we utilized medium substitution gelatin methacrylate (GelMA, 55–65%) as the primary component of the mixed bioinks, which helped achieve a balance between mechanical strength and cell compatibility. The rapid crosslinking advantages with 405 nm blue light under the photo-initiator lithium phenyl-2,4,6-trimethylbenzoylphosphinate (LAP), along with excellent 3D printing performance, have rendered this material one of the most commonly used materials in 3D bioprinting. However, the relatively inferior mechanical properties of GelMA cannot fulfill the mechanical requirements of tissue engineering [27]. To address this issue, a considerable number of articles have been published on incorporating inorganic or organic particles to enhance the mechanical properties of GelMA and regulate the rate of GelMA degradation. For instance, Gao *et al.* [28] added polyN-acryll glycine (PACG) to GelMA and demonstrated that the inclusion of PACG improved the compression and tensile strength of GelMA. Similarly, Gao *et al.* [27] showed that the addition of nanoclays to GelMA improved the material's porosity, enhanced its mechanical strength, reduced its degradation rate and maintained its excellent biocompatibility.

Chitin is known to exist in two main crystal forms: α crystal form and β crystal form. The molecular chains of α-chitin are arranged in an anti-parallel manner, which is conducive to the formation of strong intermolecular hydrogen bonds. In contrast, the molecular chains in β-chitin are arranged in parallel. α-Chitin exists in the exoskeletons of various arthropods, such as shrimp, crabs and other insects, while β-chitin is less abundant and is found in squid pen and cuttlefish bones. Due to its wide range of sources and stable structure, we chose α-chitin as the experimental material. Chitin nanocrystal (ChiNC) is an organic nanocrystal obtained from chitin acid solution. It is also a natural, non-toxic material with cell compatibility and has been shown to enhance the mechanical properties of various materials [29]. For example, Gu *et al.* [30] found that ChiNC could regulate the natural polymer flow behavior and act as an organic nanofiller to improve the scaffold's mechanical properties. Similarly, Liu *et al.* [31] discovered that ChiNC could increase the stress resistance of poly(L-lactide), facilitating cell adhesion, proliferation and upregulation of alkaline phosphatase activity, promoting calcium deposition. Hassanzadeh *et al.* [32] added ChiNC to GelMA to enhance its mechanical properties, this study showed that the addition of ChiNC increased the elastic modulus of GelMA by 1000 times and the strain by more than 200%. However, this study did not integrate 3D bioprinting technology and did not include a comprehensive investigation of vascularization.

In this study, we investigated the influence of incorporating various concentrations of ChiNC into GelMA on the physical and chemical properties of GelMA. In conjunction with 3D bioprinting, human umbilical vein endothelial cells (HUVECs) were seeded into the GelMA/ChiNC bioink. Utilizing 3D bioprinting technology, we subsequently fabricated a cell-laden scaffold, enabling us to examine the adhesion, proliferation, printability and vascularization expression of HUVECs within hydrogel scaffolds. Moreover, we assessed the 3D printing fidelity of both the 0% ChiNC group and the 1% ChiNC group at varying temperatures.

## Materials and methods

### Preparation and characterization of the ChiNC

ChiNC was prepared with slight modifications according to the method described by Wang [33]. First, 3 mol/l hydrochloric acid solution was added to chitin, which was then placed on a magnetic stirrer and heated to 60°C while stirring at a speed of 300 r/min for 6 h. The residue was washed with deionized water and centrifuged. The obtained suspension was filtered through a 100-mesh sieve and transferred to a dialysis bag (12KD–14KD) for dialysis in deionized water for 48 h. After dialysis, the suspension was sonicated (Scientz-950E, China) at 100 W of power for 20 min and then freeze-dried using a freeze-drying machine (SCIENTZ-10N, China) to obtain ChiNC powder. The resulting ChiNC powder was sterilized by cobalt-60 irradiation. The ChiNC were redispersed in a solution using an ultrasonic machine before use. We characterized ChiNC using transmission electron microscopy (TEM, JEM-F200, Japan). Using a pipette, 100 μl of ChiNC suspension was dropped onto a 230-mesh carbon, naturally dried and then the sample was observed and photographed under a TEM. The images were processed using Image J, and the length frequency distribution of ChiNC was analyzed using GraphPad Prism 8.0.2 software.

### Bioink preparation

Different concentrations (0%, 0.5%, 1% and 2%) of ChiNC were added to 10% (w/v) GelMA (SP-BI-G01-4, SunP Biotech, China) + 0.25% (w/v) LAP (SP-BI-C02-2, SunP Biotech, China) bioinks, referred to as 0%, 0.5%, 1% and 2% ChiNC bioinks, where 0% denoted no ChiNC. GelMA and LAP were first dissolved in phosphate-buffered saline (PBS, Gibco, USA) at 60°C, filtered and sterilized using a 0.22-micron filter. GelMA/LAP bioinks with varying concentrations of ChiNC (0%, 0.5%, 1% and 2% ChiNC groups) were then prepared by adding various amounts of sterile ChiNC to GelMA/LAP bioinks and sonicated at 100 W of power for 10 min before use.

### Hydrogel porosity

Four bioinks (0%, 0.5%, 1% and 2% ChiNC) were cured with 405 nm blue light for 2 min, lyophilized in a cold lyophilizer (SCIENTZ-10N, China) and the lateral pores were observed by cutting the samples along the longitudinal section. The samples were attached to sample brackets, and after the samples were coated with gold, the microscopic pores were observed using a scanning electron microscope (SEM, QUANTA FEG 250, USA). Porosity was defined as the ratio of the surface area occupied by the pores in the SEM images and analyzed using Image J software 1.53c. The data were statistically analyzed and graphed using GraphPad Prism 8.0.2 software.

### Hydrogel equilibrium swelling rate

Four bioinks (0%, 0.5%, 1% and 2% ChiNC) were added to 48-well plates with four replicate wells for each group (500 μl per well). These structures were exposed to UV light (405 nm) for 5 min and freeze-dried for 48 h, after which the dry weight of each sample was recorded (Wd). The samples were then soaked in 1.5 ml PBS at 37°C for 48 h. Finally, we gently wiped the PBS on the sample surface with filter paper and recorded the weight (Ws) of each sample. The equilibrium swelling rate was calculated using the formula (Ws−Wd)/Wd.

### Hydrogel rheological properties

The rheological properties of hydrogels were tested using a rheometer (Anton Paar MCR 302, AntonPaar, Australia) with a parallel-plate geometry (25 mm in diameter, a gap height of 50 µm). The rheometer assessed the shear rate and viscosity of the four bioinks (0%, 0.5%, 1% and 2% ChiNC) in the shear rate range of 10^0^–10^3^ (s^−1^) at a temperature of 37°C. The storage modulus versus frequency was tested at 0.5% strain, a frequency range of 10^−1^–10^1^ (Hz) and at 37°C.

### Hydrogel mechanical properties

Four bioinks (0%, 0.5%, 1% and 2% ChiNC) were prepared and cured using 405 nm blue light for 5 min to form cylindrical hydrogels with a diameter of 12.6 mm and a thickness of 9.5 mm. The stress-strain behavior of each sample was measured using a universal mechanical compressor (EUT6502, SASTest, China) at a temperature of 25°C and a compression speed of 0.1 mm/s. Three replicates were assessed for each of the four groups. The differences in fracture strain among the four hydrogel groups were analyzed using GraphPad Prism software.

### Cell culture

HUVECs (CTCC-0804-PC, China) were cultured in specialized endothelial cell culture medium (ECM, CTCC-002-031, China) containing 10% fetal bovine serum (Gibco, China) and 1% penicillin-streptomycin (SV30010, HyClone, Germany). The HUVECs were cultured in 100 mm diameter cell culture dishes within a cell incubator at 37°C and 5% CO_2_, with the culture medium being replaced every 2 days. Cells were passaged using 0.05% Trypsin-EDTA (59417C-500ML, Sigma-Aldrich, USA), and the third to sixth passages of HUVECs were used in our experiments.

### Cell viability

HUVECs were added to four groups of bioinks (0%, 0.5%, 1% and 2% ChiNC), with a cell density of 8 × 10^6^ cells/ml. 3D bioprinted grid scaffolds were cultured in ECM, and cell viability within the 3D bioprinted scaffolds was assessed using a fluorescent live/dead activity assay kit (KGAF001, KeyGEN Bio tech, China) on Days 1 and 7 according to the protocol. The scaffolds were visualized under a fluorescence microscope (NIKON Ti-A1, Japan), and images were processed with Image J software.

### Cell proliferation

Four groups of bioinks (0%, 0.5%, 1% and 2%) were added to a 48-well plate, each group consisted of four replicate wells, to which 200 μl of hydrogel was added. The hydrogel in each well was cured using 405 nm blue light for 2 min. Subsequently, 2 × 10^5^ HUVECs were seeded on the surface of each hydrogel, and 600 μl of culture medium was added to each well. The samples were incubated at 37°C in 5% CO_2_, with the culture medium being replaced every 2 days. On Days 1, 3 and 7, the samples from the different groups were washed with PBS three times. Following this, 180 μl of ECM culture medium and 20 μl of Cell Counting Kit-8 solution (CCK-8, Dojindo, Japan) were added to each well. After a 2-h incubation period, the samples were transferred to a 96-well plate (100 μl/well), and the absorbance [optical density (OD) 450 nm] was measured using a microplate photometer (VersaMax, USA).

### Immunofluorescence staining

HUVECs were cultured on the surface of ‘10% GelMA + 1% ChiNC’ hydrogel scaffolds. Immunofluorescence staining for CD31 was performed on Days 7 and 14 according to the manufacturer's protocol. Three random fields were selected and photographed, and the images were analyzed using Image J software.

### Enzyme-linked immunosorbent assay

HUVECs were seeded in the four scaffold groups, and culture supernatants were collected on Days 3 and 7. The concentrations of vascular endothelial growth factor (VEGF) and basic fibroblast growth factor (bFGF) in the culture supernatant were measured using VEGF (PV963, Cloud-Clone, China) and bFGF enzyme-linked immunosorbent assay (ELISA) kits (ab246531, Abcam, UK), respectively, following the manufacturer's protocol. This allowed for the evaluation of the impact of ChiNC concentration on the secretion of vascular secretory factors from HUVECs.

### Comparison of bioink printing effects

To better observe the printing effect of bioink, we added a 0.5% (v/v) concentration of pigment (FLEUR COULEUR, China) to the bioink in both the 0% ChiNC and 1% ChiNC group. The printing effects of bioinks (0% ChiNC and 1% ChiNC groups) were evaluated at temperatures between 22°C and 29°C. In this study, we adjusted the printing temperature and allowed a 10-min waiting period each time to ensure accurate printing effects before testing. All scaffolds were printed with four layers and had dimensions of 15 mm × 15 mm × 1.0 mm.

### 3D Bioprinted GelMA/ChiNC scaffold

In this study, scaffolds were printed using a bioprinter (SunP BioMaker 4, China) under sterile conditions throughout the entire bioprinting process. The interior of the 3D printer was wiped with gauze soaked in 75% alcohol, and the printer's ultraviolet sterilization system was activated for 30 min. HUVECs were resuspended in 37°C hydrogels at a density of 8 × 10^6^ cells/ml and loaded into a 3D printer syringe.

The printing parameters for live/dead staining scaffolds were as follows: the inner diameter of the printing needle was 0.25 mm, the line spacing was 3.0 mm, the printing nozzle temperature was set to 25°C, and the cooling platform was maintained at 8°C. The printing speed was 2 mm/s, with an extrusion speed of 0.3 mm^3^/s. Blue light irradiation at 50% intensity was used, with a distance of 60 mm and a duration of 30 s.

### Statistical analysis

All data were expressed as mean ± standard deviation (SD). Statistical analysis was performed using ANOVA with GraphPad Prism 8.0.2 (GraphPad Software, USA), where *P* < 0.05 was considered statistically significant. The data were indicated with * for *P* < 0.05, ** for *P* < 0.01, *** for *P* < 0.001, **** for *P* < 0.0001 and NS for no statistical significance.

## Results

### Preparation and characterization of ChiNC

**Figure 1.**
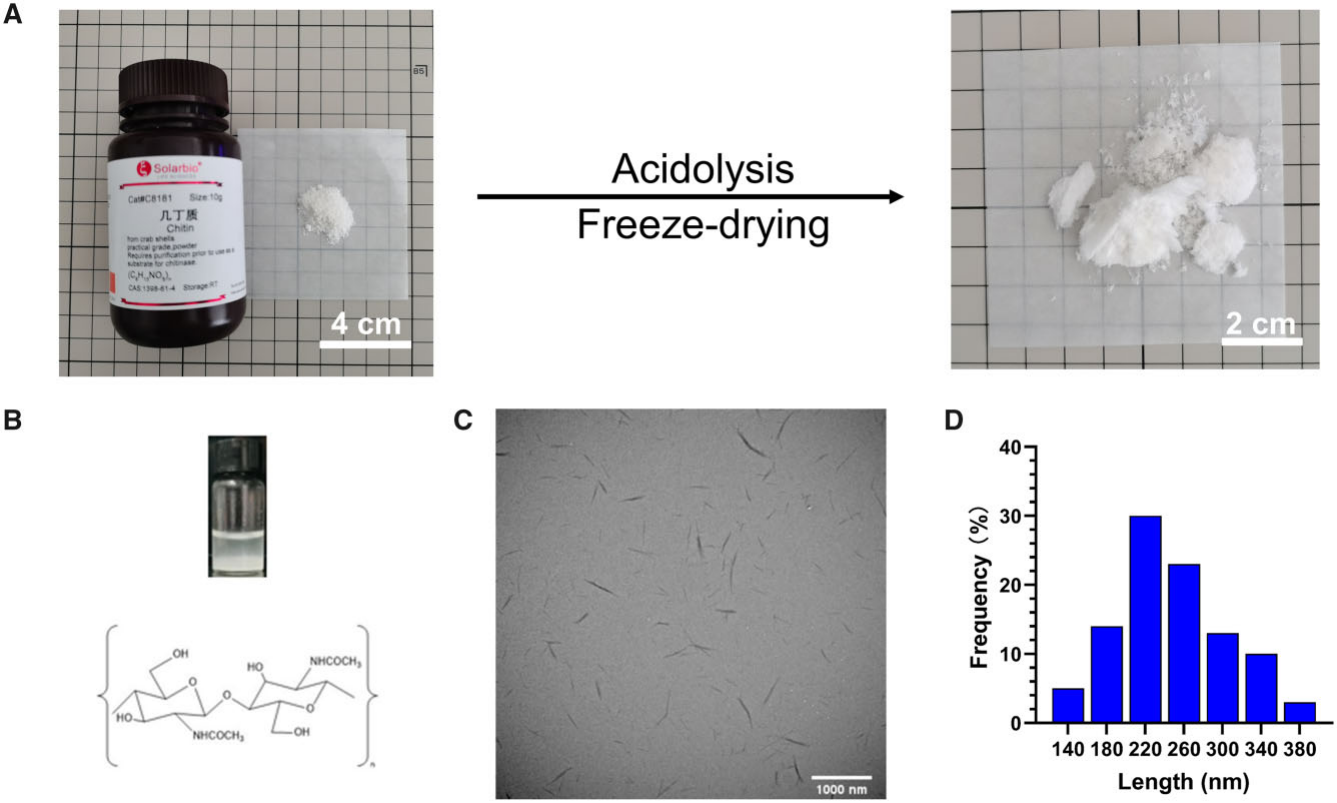
Preparation and characterization of ChiNC. (**A**) ChiNC obtained through the acidolysis of chitin with hydrochloric acid and subsequent freeze-drying. (**B**) Optical image of ChiNC aqueous suspension and the structural formula of ChiNC. (**C**) TEM image of ChiNC in the aqueous suspension. (**D**) Length/frequency distribution of ChiNC.

ChiNC, a rod-like nanowhisker, was prepared by acidolysis of chitin with hydrochloric acid, followed by lyophilization (Fig. 1A). The aqueous solution of ChiNC appears as a milky emulsion, and its molecular structure indicates that it is a polysaccharide (Fig. 1B). The TEM image of ChiNC shows that it resembles a rod-like whisker (Fig. 1C), with an average length of 245.94 ± 57.75 nm (Fig. 1D). Most of the lengths were concentrated in the range of 160–320 nm (81.63%), with the different length ranges as follows: 160–200 nm (14.29%), 200–240 nm (30.61%), 240–280 nm (23.47%) and 280–320 nm (13.27%).

### Characterization of GelMA/ChiNC bioinks

**Figure 2.**
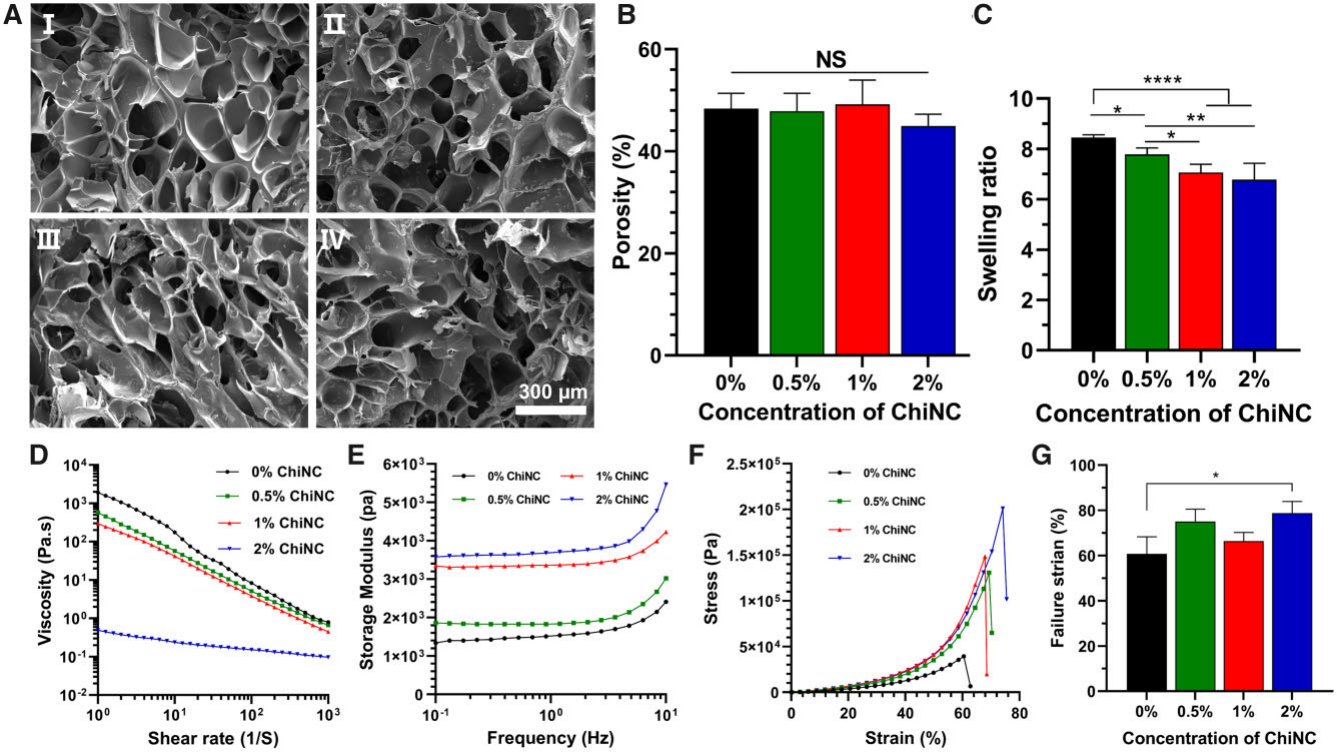
Characterization of the GelMA/ChiNC hydrogel. (**A**) SEM cross-sectional images (aI: 0% ChiNC, aII: 0.5% ChiNC, aIII: 1% ChiNC and aIV: 2% ChiNC), (**B**) porosity, (**C**) equilibrium swelling ratio, (**D**) viscosity–shear rate relationship, (**E**) storage modulus–angular frequency relationship, (**F**) compressive stress–strain curves and (**G**) failure strain of the 10% GelMA with various concentrations of ChiNC bioinks.

We prepared four groups of bioinks with varying concentrations of ChiNC (0%, 0.5%, 1% and 2% ChiNC) and cured them with blue light and lyophilization. The cross-sections of the hydrogels were examined under SEM (Fig. 2A), and the porosity of the four groups of hydrogels was ∼50%, with no statistically significant differences observed (Fig. 2B).

The addition of 2% ChiNC to 10% GelMA reduced the average equilibrium swelling ratio of the hydrogel from 8.451–6.775. The 10% GelMA alone exhibited a statistically significant difference compared to the other three groups (Fig. 2C).

To assess whether the GelMA/ChiNC bioinks could be extruded for 3D bioprinting, we evaluated the shear rate–viscosity and storage modulus–angular frequency of the four groups of bioinks. As depicted in Fig. 2D, higher shear rates resulted in lower viscosities. Under the same conditions, the viscosity of the bioinks decreased with the addition of ChiNC. The incorporation of 1% ChiNC into 10% GelMA reduced the viscosity to 46.06% at a shear rate of 100 (1/s). Moreover, the viscosity significantly decreased to 1.81% when the ChiNC concentration reached 2%.

The storage modulus of the GelMA/ChiNC hydrogel increased with increasing angular frequency, with higher ChiNC concentrations resulting in a greater storage modulus. When the angular frequency was 10 Hz, and 2% ChiNC was added to 10% GelMA, the storage modulus increased from 2410 to 5460 Pa, a 126.6% increase (Fig. 2E). The addition of ChiNC improved the mechanical properties of the GelMA-based printed scaffold.

The stress of the hydrogel increased as the strain increased. A comparison of four different hydrogels showed that higher concentrations of ChiNC resulted in greater stress for the hydrogel, and when 2% ChiNC was added, the hydrogel's maximum stress reached 2.0 × 10^5^ Pa (Fig. 2F).

As illustrated in Fig. 2G, the addition of ChiNC increased the fracture strain of the hydrogel. Comparing the 0% ChiNC group to the 2% ChiNC group, there were statistically significant differences in fracture strain. However, the differences were not statistically significant between the 0% ChiNC group and the other groups.

### The biocompatibility of GelMA/ChiNC hydrogel

**Figure 3.**
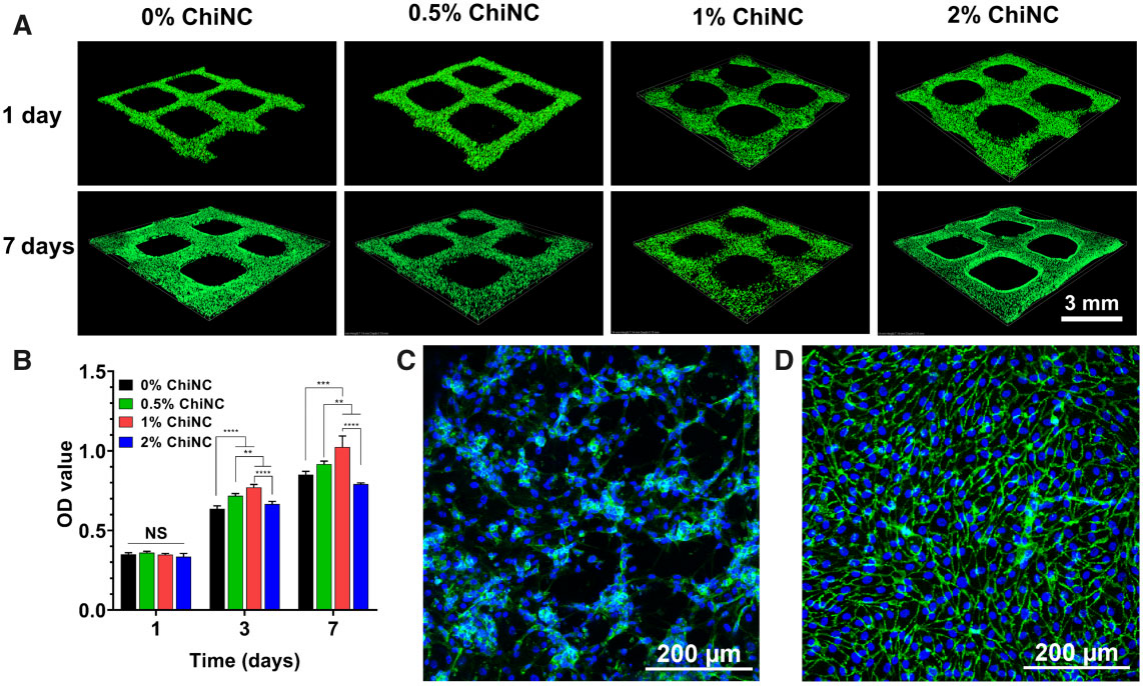
Biocompatibility of the hydrogels. (**A**) Live/dead staining of hydrogels with varying ChiNC concentrations on Days 1 and 7. (**B**) Cell Counting Kit-8 assay for hydrogels with different ChiNC concentrations on Days 1, 3 and 7 (*n* = 4). CD31 immunofluorescence staining of HUVECs on Day 7 (**C**) and Day 14 (**D**) on the surface of the 1% ChiNC group hydrogel (blue represents the nucleus and green represents CD31).

As shown in Fig. 3A, the majority of cells in each group of hydrogels were viable HUVECs, indicating good biocompatibility.

To further investigate the effects of ChiNC on the hydrogel, four types of hydrogel surfaces were cultured with HUVECs, and CCK-8 metabolic activity was assessed on Days 1, 3 and 7. There was no statistical difference between the four groups on Day 1. However, on Days 3 and 7, the OD value of the 1% ChiNC group was significantly higher than that of the other three groups (Fig. 3B).

The HUVECs grown on the hydrogel surface (10% GelMA + 1% ChiNC) were immunofluorescence stained with CD31 on Days 7 (Fig. 3C) and 14 (Fig. 3D). There was a significant increase in the number of cells on Day 14 compared to Day 7, with the cells forming tight junctions and expressing a considerable amount of CD31.

### Detection of vascularization factors in GelMA/ChiNC hydrogel

HUVECs were implanted in four groups of scaffolds, and the levels of VEGF and bFGF were measured using ELISA kits.

**Figure 4.**
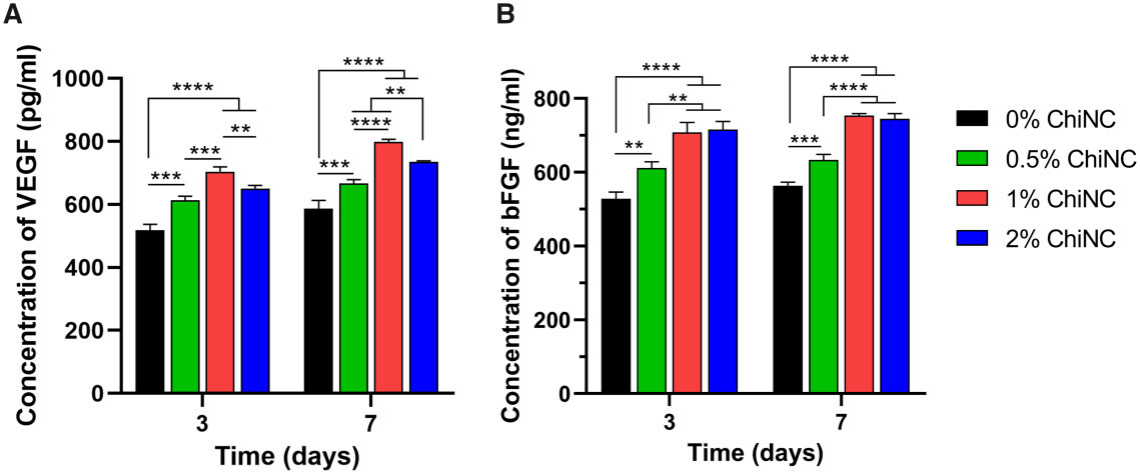
Secretion of angiogenic factors from human umbilical vein endothelial cells. Concentration of VEGF (**A**) and bFGF (**B**) in the HUVECs culture medium supernatant of GelMA hydrogels containing different concentrations of ChiNC on Days 3 and 7 (*n* = 3).

At Days 3 and 7, the 1% ChiNC group showed the highest levels of VEGF, with 704.92 ± 14.55 and 798.74 ± 8.58 pg/ml, respectively, exhibiting statistically significant differences compared to the other three groups (Fig. 4A).

The content of bFGF in the 1% ChiNC group was higher than that in the 0% ChiNC and 0.5% ChiNC groups at Days 3 and 7, with statistical significance. However, there was no statistically significant difference between the 2% ChiNC and 1% ChiNC groups at Days 3 and 7 (Fig. 4B).

### Comparison of bioink printing effects

**Figure 5.**
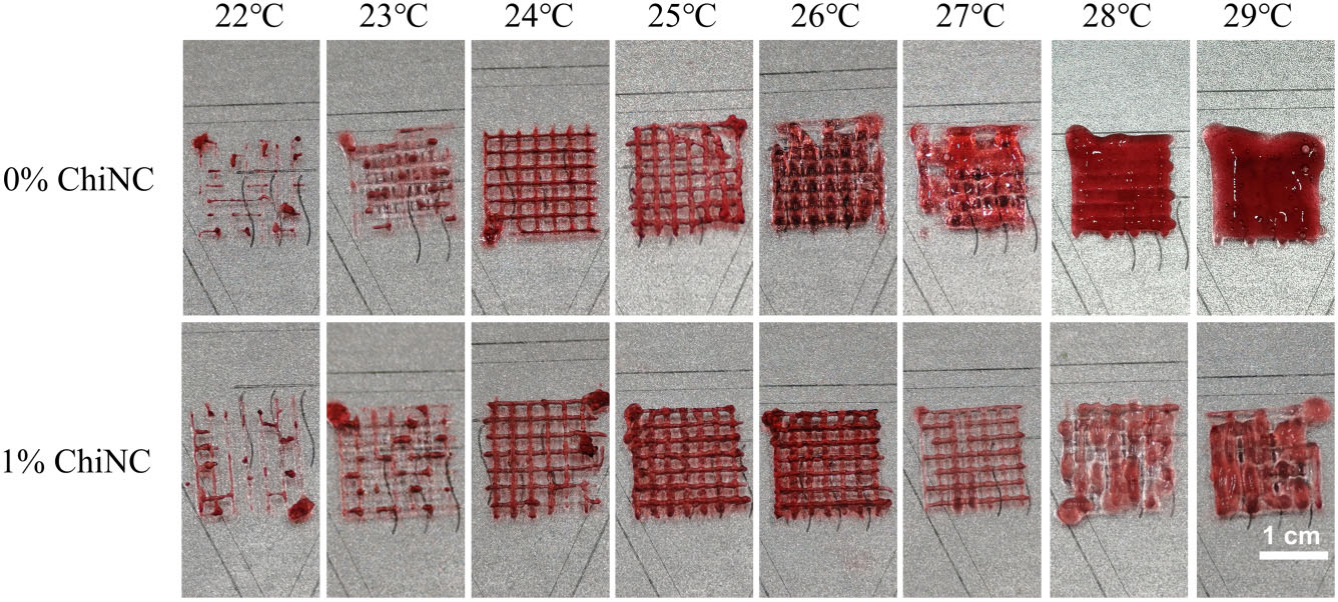
Comparison of printing effects between 0% ChiNC group and 1% ChiNC group at different temperatures.

In this study, the printing effects of two groups of bioinks were compared at different bioink temperatures. The scaffolds were printed in four layers. The printing process for the 0% ChiNC group was discontinuous and irregular at temperatures ranging from 22°C to 23°C, indicating excessive viscosity. At temperatures ranging from 24°C to 25°C, the printing was continuous and filamentary. The adjacent printed filaments fused at temperatures ranging from 26°C to 27°C. Conversely, the printing process for the 1% ChiNC group was discontinuous and irregular at temperatures ranging from 22°C to 23°C, but the printing was continuous, and filamentation was favorable at temperatures ranging from 24°C to 27°C. The adjacent printed filaments fused at temperatures ranging from 28°C to 29°C (Fig. 5).

### GelMA/ChiNC printed scaffolds

**Figure 6.**
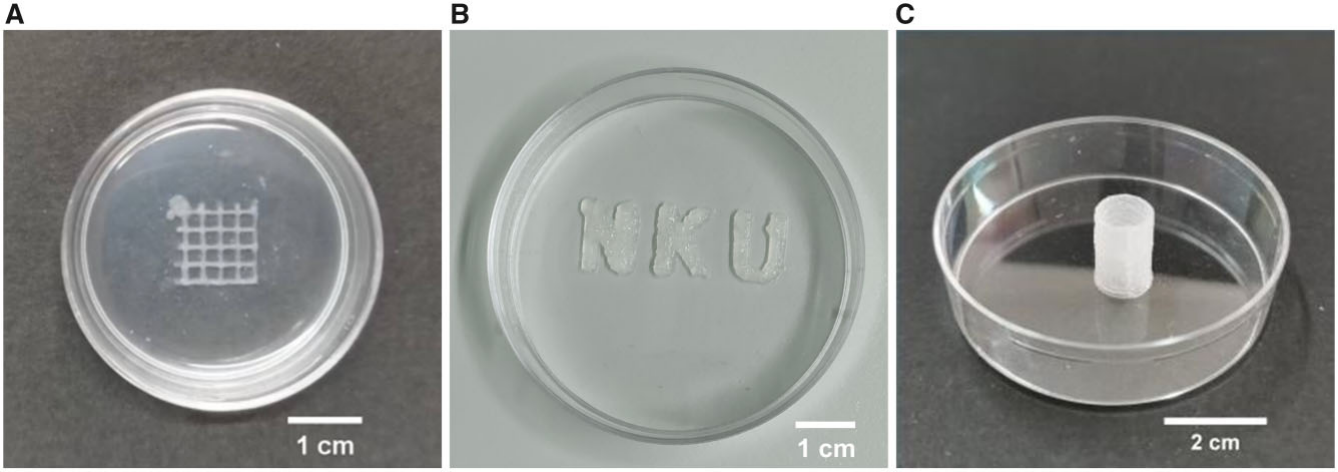
3D Printed various shaped scaffolds. 3D printed hydrogel scaffolds with a grid-like structure (**a**), 3D ‘NKU’ character structure (**B**) and tubular structure (**C**).

All structures were printed using 1% ChiNC group hydrogels. Fig. 6A illustrates the printed grid scaffold with a printed filament spacing of 2.2 mm and dimensions of 10 mm (length) × 10 mm (width) × 1.0 mm (thickness). The layer height was set at 0.25 mm, and four layers were printed. Fig. 6B presents the printed ‘NKU’ characters (the English abbreviation of Nankai University), with dimensions of 30 mm (length) × 10 mm (width) × 1.0 mm (thickness), and four layers were printed. Fig. 6C depicts the printed tube with a diameter of 10 mm and a layer height of 0.25 mm. A total of 80 layers were printed.

## Discussion

Hydrogels exhibiting excellent biocompatibility, mechanical properties, and suitability for cell proliferation have emerged as a prominent research focus [34–36]. In this study, we incorporated varying concentrations of ChiNC into 10% GelMA to examine the influence of ChiNC concentration on the rheological and mechanical properties of the bioink. Furthermore, we assessed the biocompatibility of GelMA/ChiNC hydrogels and the impact of ChiNC on the adhesion, proliferation and vascularized secretions of HUVECs within the GelMA/ChiNC hydrogels. Our findings suggest that ChiNC enhanced the mechanical properties of GelMA, reduced hydrogel viscosity and improved 3D bioprinting efficiency. Additionally, ChiNC promotes HUVECs' adhesion, proliferation and expression of vascularization factors on the hydrogel surface.

The primary sources of ChiNC raw materials are the shells of crustaceans, such as shrimp and crabs. ChiNC nanoparticles typically exhibit a whisker-like shape, with diameters ranging from 160–320 nm, in accordance with the findings of Jung *et al.* [37]. These whisker-shaped nanoparticles function analogously to the addition of steel reinforcement in concrete, bolstering the overall mechanical properties of the GelMA hydrogel.

Our study demonstrated that the incorporation of ChiNC into 10% GelMA did not alter the hydrogel's porosity, which is in line with previous research [30, 31]. Maintaining consistent porosity in hydrogels is advantageous for tissue engineering scaffolds, as it facilitates nutrient transport, cell attachment, migration and proliferation [38, 39].

As a nanomaterial, the addition of ChiNC to GelMA hydrogel led to a decreased swelling rate, a finding corroborated by previous research on GelMA [40]. With increasing ChiNC concentration, both the stiffness and interaction forces between ChiNC and GelMA rose, thereby inhibiting the swelling of GelMA/ChiNC hydrogels [29]. The diminished swelling rate of the hydrogel is advantageous for preserving the scaffold's shape by minimizing the deformation of the printed scaffold in aqueous environments.

The shear-thinning property of a hydrogel is a crucial determinant of its suitability for 3D bioprinting. Our findings revealed that the viscosity of GelMA hydrogel diminished as the shear rate increased, a result consistent with numerous studies [41–43]. A decrease in viscosity during extrusion through the print nozzle is beneficial, while viscosity recovery post-printing on the platform is favorable for retaining the original shape. Consequently, the shear-thinning characteristic of the hydrogel is advantageous for 3D bioprinting. Introducing ChiNC to 10% GelMA decreased the hydrogel's viscosity, with a substantial reduction in the viscosity of GelMA/ChiNC hydrogel observed at a ChiNC concentration of 2%. As printed scaffolds can deform more easily when the viscosity of the mixed hydrogel is too low, we think that the 2% ChiNC group bioink is unsuitable for 3D bioprinting.

Hydrogel scaffolds require good mechanical properties, including good elastic properties and mechanical strength to be suitable for *in vivo* transplantation. The storage modulus is an indicator of elasticity, and our results demonstrate that the incorporation of ChiNC increases the storage modulus of the hybrid hydrogel, this is due to the physical cross-linking between GelMA and ChiNC, resulting in an improvement in the storage modulus of the hydrogel network. A higher storage modulus enables the hydrogel to retain its original shape better, thus improving printing reliability [44, 45]. The increased elasticity of the hydrogel is beneficial for tissue engineering scaffolds.

We also noted that the mechanical strength of the hydrogels was substantially enhanced by incorporating ChiNC into 10% GelMA, with increased ChiNC concentrations resulting in higher stress in the hybrid hydrogels under identical strain. This phenomenon can be attributed to the high aspect ratio and modulus of ChiNC, which enables the dispersion of greater pressure loads for GelMA, as well as the bridging role of nanoparticles within the hydrogel network, improving network stability and stress resistance [31, 46]. This finding also elucidates the reason behind the improvement in the failure strain rate of the hybrid hydrogels upon ChiNC addition, as enhanced compression resistance would be beneficial for tissue engineering applications.

Biocompatibility is a fundamental criterion for biomaterials used in tissue engineering, as it underpins cell adhesion, growth and proliferation on hydrogel scaffolds. In this study, we assessed the biocompatibility of GelMA/ChiNC hydrogels by seeding HUVECs within the hydrogel and cultivating them for 7 days. We conducted live/dead staining, which demonstrated that the cells thrived within the hydrogel, Live/dead staining revealed that the cells exhibited high survival rates within the hydrogel, suggesting that the GelMA/ChiNC hydrogel is biocompatible and appropriate for bioink printing. Furthermore, we seeded HUVECs on the hydrogel surface and assessed cell metabolic viability using the CCK-8 assay. The CCK-8 results demonstrated no statistically significant differences in OD values among the four groups on Day 1. The OD values increased with the rising ChiNC concentration on Days 3 and 7, whereas they decreased when the ChiNC concentration reached 2%. This observation suggests that low concentrations of ChiNC might promote cell adhesion and proliferation. However, at a ChiNC concentration of 2%, the hydrogel surface morphology appeared unfavorable for cell adhesion and proliferation [47].

Since HUVECs express the endothelial cell-specific antigen CD31, a marker of vascularization, we used CD31 immunofluorescence staining to assess vascularization. The CD31 immunofluorescence results revealed that HUVECs adhered and grew effectively on the ‘10% GelMA + 1% ChiNC’ hydrogel surface, further demonstrating the suitability of ‘10% GelMA + 1% ChiNC’ hydrogel for cell adhesion and proliferation.

One of the challenges in tissue engineering is the absence of optimal vascularization. Presently, the incorporation of angiogenic growth factors such as VEGF and bFGF serves as a potential solution to promote vascularization. Both VEGF and bFGF exhibit a synergistic effect in stimulating angiogenesis [48–51]. Enhanced expression of these factors can prompt endothelial cells to degrade the basement membrane, enabling them to migrate to the defective site for proliferation. Eventually, new vessel lumens emerge, the capillary network becomes denser and new vessels mature [52]. Thus, we assessed the expression of VEGF and bFGF vascularization factors to ascertain the role of ChiNC in promoting vascularization. In our study, we seeded HUVECs into four hydrogel groups with varying concentrations of ChiNC (0%, 0.5%, 1% and 2%) to examine the effect of ChiNC on the secretion of angiogenic growth factors by HUVECs. Our results indicated that VEGF secretion increased with the rising concentration of ChiNC but decreased when the concentration reached 2%. This observation was consistent with the CCK-8 results, which suggested that the increase in cell number may have contributed to the elevated VEGF secretion. However, other factors, such as differences in gene expression, could have also played a role. Similarly, our study revealed that bFGF secretion increased with escalating ChiNC concentration but ceased to increase when the concentration reached 2%. Although, theoretically, a decline in cell metabolic viability at 2% ChiNC should have led to a reduction in bFGF secretion, we did not observe this phenomenon. It is plausible that differences in gene expression accounted for this observation, warranting further investigation.

Printability is a fundamental prerequisite for hydrogels intended for use as bioinks. In this study, we assessed the physical properties, biocompatibility and ELISA results of four hydrogel groups to identify the most appropriate bioink for 3D bioprinting. Our findings indicate that the 1% ChiNC hydrogel is a superior candidate for 3D bioprinting due to its broader printable window compared to the control group (0% ChiNC). Incorporating nanoparticles into the GelMA hydrogel enhanced the hydrogel network's stability, improved its storage modulus and broadened the 3D bioprinting range.

We used the ‘10% GelMA + 1% ChiNC’ bioink for 3D bioprinting of grids, ‘NKU’ characters and tubes, suggesting that this bioink is a viable option for 3D bioprinting applications. Our bioink exhibited favorable biocompatibility, mechanical properties, cell proliferation and vascularization promotion and enhanced 3D printing outcomes. Consequently, we believe it may be applicable in various tissue engineering contexts, such as bladder and skin tissue engineering.

Currently, we are conducting further research on hydrogels based on GelMA and ChiNC for bladder defect repair. We posit that incorporating ChiNC into hydrogels could augment their efficacy and broaden their application scope. This strategy might also be applicable to other types of bioinks in tissue engineering. In conclusion, our study highlights the potential of our bioink for 3D bioprinting and its prospective applications in tissue engineering.

## Conclusions

This study investigated the effects of different concentrations of ChiNC on 10% GelMA hydrogel concerning mechanical properties, rheological properties, porosity, equilibrium swelling rate, biocompatibility, cell adhesion and proliferation. Our findings demonstrated the impact of ChiNC addition to GelMA on cell adhesion and proliferation. The results revealed that incorporating ChiNC into GelMA enhanced the mechanical properties, printability, cell adhesion and proliferation. ELISA results of angiogenic factors and 3D printing indicated that the 1% ChiNC group was optimal for 3D bioprinting. Thus, we recommend the 1% ChiNC group as the most suitable for 3D bioprinting due to its improved printability, cell support and proliferation. Furthermore, the addition of ChiNC could be explored in conjunction with other bioinks to enhance their application potential in tissue engineering. In conclusion, ChiNC proves to be a promising biomaterial in the field of tissue engineering.

## References

[1] Gong J , Schuurmans C, Genderen A, Cao X, Li W, Cheng F, He JJ, Lopez A, Huerta V, Manriquez J, Li R, Li H, Delavaux C, Sebastian S, Capendale PE, Wang H, Xie J, Yu M, Masereeuw R, Vermonden T, Zhang YS. Complexation-induced resolution enhancement of 3D-printed hydrogel constructs. Nat Commun 2020;11:1267. 32152307 10.1038/s41467-020-14997-4 PMC7062888

[2] Jeon O , Lee YB, Lee SJ, Guliyeva N, Lee J, Alsberg E. Stem cell-laden hydrogel bioink for generation of high resolution and fidelity engineered tissues with complex geometries. Bioact Mater 2022;15:185–93. 35386348 10.1016/j.bioactmat.2021.11.025 PMC8940765

[3] Van Belleghem S , Torres LJ, Santoro M, Mahadik B, Wolfand A, Kofinas P, Fisher JP. Hybrid 3d printing of synthetic and cell-laden bioinks for shape retaining soft tissue grafts. Adv Funct Mater 2020;30:1907145. 33041744 10.1002/adfm.201907145 PMC7546434

[4] Wu Y , Liang T, Hu Y, Jiang S, Luo Y, Liu C, Wang G, Zhang J, Xu T, Zhu L. 3D bioprinting of integral ADSCs-NO hydrogel scaffolds to promote severe burn wound healing. Regen Biomater 2021;8:rbab014. 33936750 10.1093/rb/rbab014 PMC8071097

[5] Correa S , Grosskopf AK, Lopez HH, Chan D, Yu AC, Stapleton LM, Appel EA. Translational applications of hydrogels. Chem Rev 2021;121:11385–457. 33938724 10.1021/acs.chemrev.0c01177 PMC8461619

[6] Below CR , Kelly J, Brown A, Humphries JD, Hutton C, Xu J, Lee BY, Cintas C, Zhang X, Hernandez-Gordillo V, Stockdale L, Goldsworthy MA, Geraghty J, Foster L, O'Reilly DA, Schedding B, Askari J, Burns J, Hodson N, Smith DL, Lally C, Ashton G, Knight D, Mironov A, Banyard A, Eble JA, Morton JP, Humphries MJ, Griffith LG, Jørgensen C. A microenvironment-inspired synthetic three-dimensional model for pancreatic ductal adenocarcinoma organoids. Nat Mater 2022;21:110–9. 34518665 10.1038/s41563-021-01085-1 PMC7612137

[7] Mosquera MJ , Kim S, Bareja R, Fang Z, Cai S, Pan H, Asad M, Martin ML, Sigouros M, Rowdo FM, Ackermann S, Capuano J, Bernheim J, Cheung C, Doane A, Brady N, Singh R, Rickman DS, Prabhu V, Allen JE, Puca L, Coskun AF, Rubin MA, Beltran H, Mosquera JM, Elemento O, Singh A. Extracellular matrix in synthetic hydrogel-based prostate cancer organoids regulate therapeutic response to EZH2 and DRD2 inhibitors. Adv Mater 2022;34:e2100096. 34676924 10.1002/adma.202100096 PMC8820841

[8] Luo X , Fong E, Zhu C, Lin Q, Xiong M, Li A, Li T, Benoukraf T, Yu H, Liu S. Hydrogel-based colorectal cancer organoid co-culture models. Acta Biomater 2021;132:461–72. 33388439 10.1016/j.actbio.2020.12.037

[9] Kaur S , Kaur I, Rawal P, Tripathi DM, Vasudevan A. Non-matrigel scaffolds for organoid cultures. Cancer Lett 2021;504:58–66. 33582211 10.1016/j.canlet.2021.01.025

[10] Amorim S , Soares DCD, Pashkuleva I, Reis CA, Reis RL, Pires RA. 3D hydrogel mimics of the tumor microenvironment: the interplay among hyaluronic acid, stem cells and cancer cells. Biomater Sci 2021;9:252–60. 33191428 10.1039/d0bm00843e

[11] Ding Z , Zhou M, Zhou Z, Zhang W, Jiang NE, Ref X, Lu X, Zuo B, Lu Q, Kaplan DL. Injectable silk nanofiber hydrogels for sustained release of small-molecule drugs and vascularization. ACS Biomater Sci Eng 2019;5:4077–88. 33448809 10.1021/acsbiomaterials.9b00621

[12] Fan C , Shi J, Zhuang Y, Zhang L, Huang L, Yang W, Chen B, Chen Y, Xiao Z, Shen H, Zhao Y, Dai J. Myocardial-infarction-responsive smart hydrogels targeting matrix metalloproteinase for on-demand growth factor delivery. Adv Mater 2019;31:e1902900. 31408234 10.1002/adma.201902900

[13] Xie M , Gao Q, Zhao H, Nie J, Fu Z, Wang H, Chen L, Shao L, Fu J, Chen Z, He Y. Electro-assisted bioprinting of low-concentration GelMA microdroplets. Small 2019;15:e1804216. 30569632 10.1002/smll.201804216

[14] Diehl F , Hageneder S, Fossati S, Auer SK, Dostalek J, Jonas U. Plasmonic nanomaterials with responsive polymer hydrogels for sensing and actuation. Chem Soc Rev 2022;51:3926–63. 35471654 10.1039/d1cs01083b PMC9126188

[15] Qin M , Sun M, Bai R, Mao Y, Qian X, Sikka D, Zhao Y, Qi HJ, Suo Z, He X. Bioinspired hydrogel interferometer for adaptive coloration and chemical sensing. Adv Mater 2018;30:e1800468. 29638026 10.1002/adma.201800468

[16] Zhao Y , Cui J, Qiu X, Yan Y, Zhang Z, Fang K, Yang Y, Zhang X, Huang J. Manufacturing and post-engineering strategies of hydrogel actuators and sensors: from materials to interfaces. Adv Colloid Interface Sci 2022;308:102749. 36007285 10.1016/j.cis.2022.102749

[17] Khajouei S , Ravan H, Ebrahimi A. DNA hydrogel-empowered biosensing. Adv Colloid Interface Sci 2020;275:102060. 31739981 10.1016/j.cis.2019.102060 PMC7094116

[18] Cao H , Duan L, Zhang Y, Cao J, Zhang K. Current hydrogel advances in physicochemical and biological response-driven biomedical application diversity. Signal Transduct Target Ther 2021;6:426. 34916490 10.1038/s41392-021-00830-x PMC8674418

[19] Vernerey FJ , Lalitha SS, Muralidharan A, Bryant SJ. Mechanics of 3D cell-hydrogel interactions: experiments, models, and mechanisms. Chem Rev 2021;121:11085–148. 34473466 10.1021/acs.chemrev.1c00046

[20] Schwab A , Levato R, D'Este M, Piluso S, Eglin D, Malda J. Printability and shape fidelity of bioinks in 3D bioprinting. Chem Rev 2020;120:11028–55. 32856892 10.1021/acs.chemrev.0c00084 PMC7564085

[21] Shapira A , Dvir T. 3D tissue and organ printing-hope and reality. Adv Sci (Weinh) 2021;8:2003751. 34026444 10.1002/advs.202003751 PMC8132062

[22] Chen C , Xu HH, Liu XY, Zhang YS, Zhong L, Wang YW, Xu L, Wei P, Chen YX, Liu P, Hao CR, Jia XL, Hu N, Wu XY, Gu XS, Chen LQ, Li XH. 3D printed collagen/silk fibroin scaffolds carrying the secretome of human umbilical mesenchymal stem cells ameliorated neurological dysfunction after spinal cord injury in rats. Regen Biomater 2022;9:c14. 10.1093/rb/rbac014 PMC9036898 35480857

[23] De Santis MM , Alsafadi HN, Tas S, Bolukbas DA, Prithiviraj S, Da SI, Mittendorfer M, Ota C, Stegmayr J, Daoud F, Konigshoff M, Sward K, Wood JA, Tassieri M, Bourgine PE, Lindstedt S, Mohlin S, Wagner DE. Extracellular-matrix-reinforced bioinks for 3D bioprinting human tissue. Adv Mater 2021;33:e2005476. 33300242 10.1002/adma.202005476 PMC11469085

[24] Lee A , Hudson AR, Shiwarski DJ, Tashman JW, Hinton TJ, Yerneni S, Bliley JM, Campbell PG, Feinberg AW. 3D bioprinting of collagen to rebuild components of the human heart. Science 2019;365:482–7. 31371612 10.1126/science.aav9051

[25] Cheng Y , Chan KH, Wang XQ, Ding T, Li T, Lu X, Ho GW. Direct-ink-write 3D printing of hydrogels into biomimetic soft robots. ACS Nano 2019;13:13176–84. 31625724 10.1021/acsnano.9b06144

[26] Jian Z , Zhuang T, Qinyu T, Liqing P, Kun L, Xujiang L, Diaodiao W, Zhen Y, Shuangpeng J, Xiang S, Jingxiang H, Shuyun L, Libo H, Peifu T, Qi Y, Quanyi G. 3D bioprinting of a biomimetic meniscal scaffold for application in tissue engineering. Bioact Mater 2021;6:1711–26. 33313450 10.1016/j.bioactmat.2020.11.027 PMC7711190

[27] Gao Q , Niu X, Shao L, Zhou L, Lin Z, Sun A, Fu J, Chen Z, Hu J, Liu Y, He Y. 3D printing of complex GelMA-based scaffolds with nanoclay. Biofabrication 2019;11:035006. 30836349 10.1088/1758-5090/ab0cf6

[28] Gao F , Xu Z, Liang Q, Li H, Peng L, Wu M, Zhao X, Cui X, Ruan C, Liu W. Osteochondral regeneration with 3D-printed biodegradable high-strength supramolecular polymer reinforced-gelatin hydrogel scaffolds. Adv Sci (Weinh) 2019;6:1900867. 31406678 10.1002/advs.201900867 PMC6685475

[29] Zeng J , He Y, Li S, Wang Y. Chitin whiskers: an overview. Biomacromolecules 2012;13:1–11. 22148591 10.1021/bm201564a

[30] Gu S , Tian Y, Liang K, Ji Y. Chitin nanocrystals assisted 3D printing of polycitrate thermoset bioelastomers. Carbohydr Polym 2021;256:117549. 33483056 10.1016/j.carbpol.2020.117549

[31] Liu K , Zhu L, Tang S, Wen W, Lu L, Liu M, Zhou C, Luo B. Fabrication and evaluation of a chitin whisker/poly(L-lactide) composite scaffold by the direct trisolvent-ink writing method for bone tissue engineering. Nanoscale 2020;12:18225–39. 32856644 10.1039/d0nr04204h

[32] Hassanzadeh P , Kazemzadeh-Narbat M, Rosenzweig R, Zhang X, Khademhosseini A, Annabi N, Rolandi M. Ultrastrong and flexible hybrid hydrogels based on solution self-assembly of chitin nanofibers in gelatin methacryloyl (GelMA). J Mater Chem B 2016;4:2539–43. 27453781 10.1039/C6TB00021E PMC4950997

[33] Wang X , Liang K, Tian Y, Ji Y. A facile and green emulsion casting method to prepare chitin nanocrystal reinforced citrate-based bioelastomer. Carbohydr Polym 2017;157:620–8. 27987970 10.1016/j.carbpol.2016.10.034

[34] Gutierrez AM , Frazar EM, X KM, Paul P, Hilt JZ. Hydrogels and hydrogel nanocomposites: enhancing healthcare through human and environmental treatment. Adv Healthc Mater 2022;11:e2101820. 34811960 10.1002/adhm.202101820 PMC8986592

[35] Choi SW , Guan W, Chung K. Basic principles of hydrogel-based tissue transformation technologies and their applications. Cell 2021;184:4115–36. 34358468 10.1016/j.cell.2021.07.009 PMC8372535

[36] Guimaraes CF , Ahmed R, Marques AP, Reis RL, Demirci U. Engineering hydrogel-based biomedical photonics: design, fabrication, and applications. Adv Mater 2021;33:e2006582. 33929771 10.1002/adma.202006582 PMC8647870

[37] Jung HS , Kim HC, Ho PW. Robust methylcellulose hydrogels reinforced with chitin nanocrystals. Carbohydr Polym 2019;213:311–9. 30879674 10.1016/j.carbpol.2019.03.009

[38] Choi E , Kim D, Kang D, Yang GH, Jung B, Yeo M, Park MJ, Lee AS, Kim K, Kim JS, Jeong JC, Yoo W, Jeon HH. 3D-printed gelatin methacrylate (GelMA)/silanated silica scaffold assisted by two-stage cooling system for hard tissue regeneration. Regen Biomater 2021;8:rbab001. 33738115 10.1093/rb/rbab001 PMC7955716

[39] Karageorgiou V , Kaplan D. Porosity of 3D biomaterial scaffolds and osteogenesis. Biomaterials 2005;26:5474–91. 15860204 10.1016/j.biomaterials.2005.02.002

[40] Xie X , Wu S, Mou S, Guo N, Wang Z, Sun J. Microtissue-based bioink as a chondrocyte microshelter for DLP bioprinting. Adv Healthc Mater 2022;11:e2201877. 36085440 10.1002/adhm.202201877 PMC11468467

[41] Wu Z , Xie S, Kang Y, Shan X, Li Q, Cai Z. Biocompatibility evaluation of a 3D-bioprinted alginate-GelMA-bacteria nanocellulose (BNC) scaffold laden with oriented-growth RSC96 cells. Mater Sci Eng C Mater Biol Appl 2021;129:112393. 34579912 10.1016/j.msec.2021.112393

[42] Li R , Zhou C, Chen J, Luo H, Li R, Chen D, Zou X, Wang W. Synergistic osteogenic and angiogenic effects of KP and QK peptides incorporated with an injectable and self-healing hydrogel for efficient bone regeneration. Bioact Mater 2022;18:267–83. 35387156 10.1016/j.bioactmat.2022.02.011 PMC8961307

[43] Guzzi EA , Bischof R, Dranseikiene D, Deshmukh DV, Wahlsten A, Bovone G, Bernhard S, Tibbitt MW. Hierarchical biomaterials via photopatterning-enhanced direct ink writing. Biofabrication 2021;13:044105. 10.1088/1758-5090/ac212f 34433148

[44] Chen Y , Sui J, Wang Q, Yin Y, Liu J, Wang Q, Han X, Sun Y, Fan Y, Zhang X. Injectable self-crosslinking HA-SH/Col I blend hydrogels for in vitro construction of engineered cartilage. Carbohydr Polym 2018;190:57–66. 29628260 10.1016/j.carbpol.2018.02.057

[45] Han J , Lei T, Wu Q. High-water-content mouldable polyvinyl alcohol-borax hydrogels reinforced by well-dispersed cellulose nanoparticles: dynamic rheological properties and hydrogel formation mechanism. Carbohydr Polym 2014;102:306–16. 24507286 10.1016/j.carbpol.2013.11.045

[46] He Y , Li Q, Chen P, Duan Q, Zhan J, Cai X, Wang L, Hou H, Qiu X. A smart adhesive Janus hydrogel for non-invasive cardiac repair and tissue adhesion prevention. Nat Commun 2022;13:7666. 36509756 10.1038/s41467-022-35437-5 PMC9744843

[47] Ding Y , Chen X, Zhou Y, Ren X, Zhang W, Li M, Zhang Q, Jiang T, Ding B, Shi D, You J. Single molecular layer of chitin sub-nanometric nanoribbons: one-pot self-exfoliation and crystalline assembly into robust, sustainable, and moldable structural materials. Adv Sci (Weinh) 2022;9:e2201287. 35355436 10.1002/advs.202201287 PMC9165516

[48] Kigel B , Rabinowicz N, Varshavsky A, Kessler O, Neufeld G. Plexin-A4 promotes tumor progression and tumor angiogenesis by enhancement of VEGF and bFGF signaling. Blood 2011;118:4285–96. 21832283 10.1182/blood-2011-03-341388

[49] Losi P , Briganti E, Errico C, Lisella A, Sanguinetti E, Chiellini F, Soldani G. Fibrin-based scaffold incorporating VEGF- and bFGF-loaded nanoparticles stimulates wound healing in diabetic mice. Acta Biomater 2013;9:7814–21. 23603001 10.1016/j.actbio.2013.04.019

[50] Gu Z , Zhang X, Li L, Wang Q, Yu X, Feng T. Acceleration of segmental bone regeneration in a rabbit model by strontium-doped calcium polyphosphate scaffold through stimulating VEGF and bFGF secretion from osteoblasts. Mater Sci Eng C Mater Biol Appl 2013;33:274–81. 25428072 10.1016/j.msec.2012.08.040

[51] Elbialy ZI , Assar DH, Abdelnaby A, Asa SA, Abdelhiee EY, Ibrahim SS, Abdel-Daim MM, Almeer R, Atiba A. Healing potential of Spirulina platensis for skin wounds by modulating bFGF, VEGF, TGF-ss1 and alpha-SMA genes expression targeting angiogenesis and scar tissue formation in the rat model. Biomed Pharmacother 2021;137:111349. 33567349 10.1016/j.biopha.2021.111349

[52] Cross MJ , Claesson-Welsh L. FGF and VEGF function in angiogenesis: signalling pathways, biological responses and therapeutic inhibition. Trends Pharmacol Sci 2001;22:201–7. 11282421 10.1016/s0165-6147(00)01676-x

