# Correction to: Chitin nanocrystal-assisted 3D bioprinting of gelatin methacrylate scaffolds

**DOI:** 10.1093/rb/rbae026

**Published:** 2024-04-08

This is a correction to: Zhengyun Ling, Jian Zhao, Shiyu Song, Shuwei Xiao, Pengchao Wang, Ziyan An, Zhouyang Fu, Jinpeng Shao, Zhuang Zhang, Weijun Fu, Shenghan Song, Chitin nanocrystal-assisted 3D bioprinting of gelatin methacrylate scaffolds, Regenerative Biomaterials, Volume 10, 2023, rbad058, https://doi.org/10.1093/rb/rbad058

The originally published version of this article required correction.

Due to the author’s clerical error, in the final image in the graphical abstract, the scale was given as 500 mm, instead of 5 mm.

This has been corrected.

